# Effects of local anesthetics on yield and differentiation of synovial mesenchymal stem cells

**DOI:** 10.1038/s41598-026-36025-z

**Published:** 2026-01-16

**Authors:** Takuya Kitamura, Kentaro Endo, Nobutake Ozeki, Hisako Katano, Mitsuru Mizuno, Yusuke Nakagawa, Hideyuki Koga, Ichiro Sekiya

**Affiliations:** 1 Center for Stem Cell and Regenerative Medicine, Institute of Science Tokyo, 1-5-45 Yushima, Bunkyo-Ku, Tokyo, 113-8510 Japan; 2 Department of Joint Surgery and Sports Medicine, Graduate School of Medical and Dental Sciences, Institute of Science Tokyo, Tokyo, Japan

**Keywords:** Mesenchymal stem cell, Local anesthetic, Lidocaine, Ropivacaine, Multipotency, Chondrogenesis, Diseases, Medical research, Stem cells

## Abstract

Human synovial mesenchymal stem cells (MSCs) demonstrate high chondrogenic capacity for regenerative medicine. While ultrasound-guided collection procedures utilize local anesthetics for patient comfort, their effects on synovial MSCs remain unclear despite their known cytotoxicity to other MSC types. This study investigated whether clinically relevant concentrations of lidocaine and ropivacaine affect synovial MSC proliferation and differentiation. Human synovial tissue from eight donors undergoing knee surgery was minced and treated for 20 min with 0.5% lidocaine, 0.2% ropivacaine, or saline control. Following enzymatic digestion, cell viability and nucleated cell yield per synovial weight were assessed immediately and after a 14-day culture expansion. Trilineage differentiation capacity was evaluated through chondrogenic pellet culture, adipogenic Oil Red O staining, and calcification Alizarin Red staining. Cell viability, nucleated cell numbers per synovium weight, and cell yield after 14-day expansion showed no significant differences between treatments. Cartilage pellet weights, Oil Red O-positive adipogenic colonies, and calcification areas remained comparable across all groups. Lidocaine or ropivacaine can be safely used during ultrasound-guided synovial tissue collection without compromising therapeutic potential. These findings support the safe clinical implementation of ultrasound-guided synovial tissue harvesting using local anesthetics, reinforcing this process as a feasible and practical platform for synovial MSC-based regenerative therapies.

**Supplementary Information:**

The online version contains supplementary material available at 10.1038/s41598-026-36025-z.

## Introduction

Mesenchymal stem cells (MSCs) have gained attention as a promising cell source for use in regenerative medicine because of their multipotency^1^, immunomodulatory capacity^2^, and tissue repair-promoting capabilities^3^. In particular, synovium-derived MSCs (synovial MSCs) can reliably yield greater cell numbers than bone marrow–derived MSCs and produce a superior cartilage matrix compared with adipose-derived MSCs^4,5^. Therefore, synovial MSCs are particularly useful in regenerative medicine for joint disorders, such as articular cartilage damage^6^, meniscal injury^7,8^, and knee osteoarthritis^9^. Clinical studies and clinical trials based on these applications are ongoing^6^; however, none have successfully identified the issues presently limiting the collection of sufficient synovial MSCs for routine clinical use.

The traditional procedure for synovial tissue collection uses arthroscopy^6–9^; however, we have recently reported the successful use of ultrasound-guided collection methods^10^ as less invasive and more cost-effective protocols that make clinical synovial MSC applications more feasible. During collection, local anesthetics are injected into the skin, subcutaneous tissue, and synovial tissue to relieve patient pain while also providing an additional measure of safety by avoiding the need for general and intravenous anesthesia. However, extensive reports now indicate that local anesthetics, such as lidocaine, can have a high degree of cytotoxicity^11–14^.

In vitro experiments using bone marrow–derived and adipose-derived MSCs have shown that lidocaine exposure can decrease cell viability^11,14^, induce apoptosis^12^, and reduce cellular ATP content^11^. In contrast, exposure to ropivacaine induces less toxicity to MSCs, while also providing longer-lasting anesthetic and analgesic effects^11–14^. However, the experiments demonstrating these effects involved direct long-term exposure of the MSCs to high concentrations of local anesthetics—conditions that are completely different from those actually used during clinical tissue collection. Furthermore, no studies have yet examined the effects of local anesthetics on synovial MSCs.

The lack of knowledge regarding the effects of local anesthetic exposure on synovial MSCs, together with the previous use of experimental conditions with no clinical relevance, prompted us to design two independent two-group comparisons using saline as a control to determine the effects of anesthetics on synovial MSCs under clinical conditions. Specifically, we aimed to clarify the effects of lidocaine and ropivacaine on the proliferative and differentiation capacities of synovial MSCs by comparing (1) a saline group vs. a lidocaine group and (2) a saline group vs. a ropivacaine group. Importantly, we maintained the duration of exposure of synovial tissue to each local anesthetic at a level equivalent to that used in clinical cases. The overall goal of this study was to obtain fundamental knowledge that can serve as a guideline for anesthetic selection during synovial tissue collection.

## Methods

### Ethics approval and patient samples

The present study was approved by the Medical Research Ethics Committee of the Institute of Science Tokyo (formerly Tokyo Medical and Dental University; approval No. M2017-142). All human study subjects provided informed consent for the use of their synovial tissues. Human synovial samples were acquired from the femoral bones at the suprapatellar pouch in the knees of 8 osteoarthritis patients (age range 51–85 years; two male and six females) who underwent total knee arthroplasty.

### Synovial tissue processing and local anesthetic exposure

**Fig. 1:**
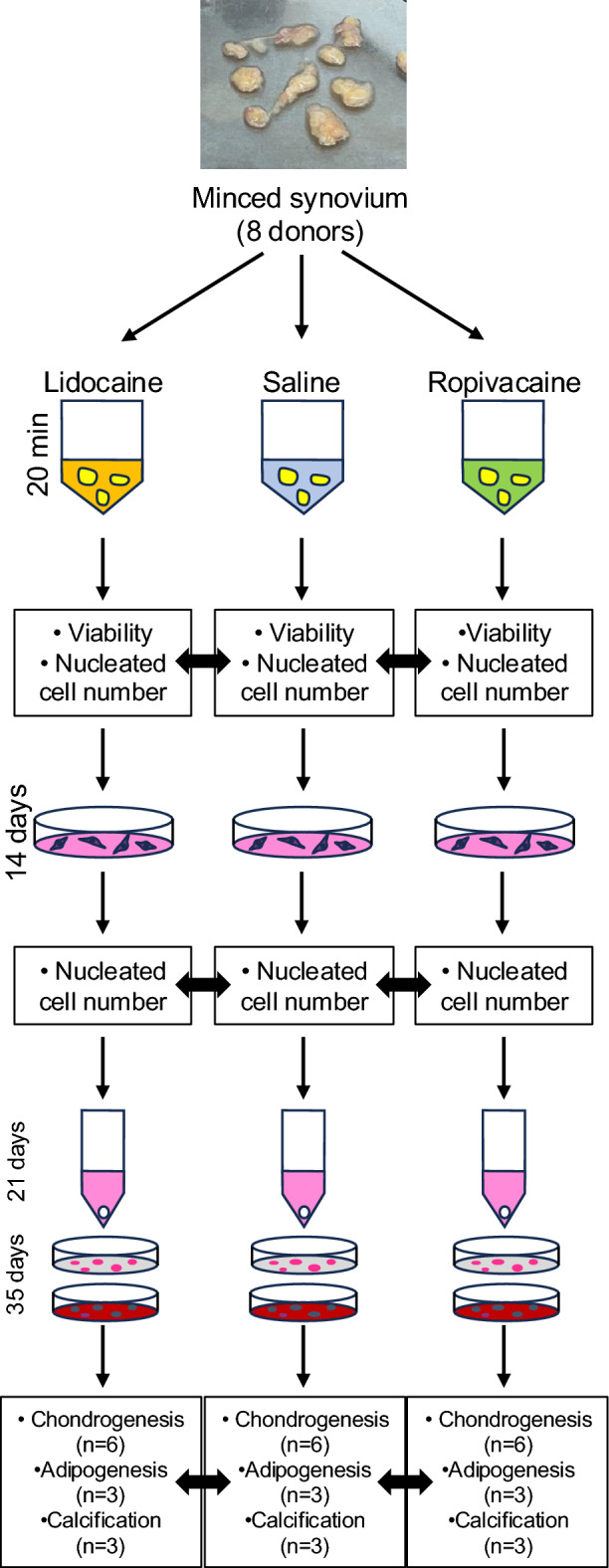
Schematic overview of the experimental design. Minced synovial tissue from eight donors was divided into three portions and exposed to lidocaine, saline, or ropivacaine, followed by enzymatic digestion and culture of the released cells. Synovial MSCs were expanded and subsequently subjected to chondrogenic, adipogenic, and calcification differentiation assays.

Approximately 1,500 mg of synovium was harvested from each patient. The tissue was minced into 8–10 fragments approximately 5 mm × 5 mm in size using a sharp rongeur and then visually divided into three equal portions. The tissue portions were weighed, allocated to different treatments, and exposed to the test solutions for 20 min at 37 °C with gentle agitation (Fig. 1). One portion was immersed in physiological saline as a control, another portion was treated with 0.5% lidocaine, and the remaining portion was exposed to 0.2% ropivacaine. The 0.5% lidocaine solution was prepared by diluting 1% lidocaine (Xylocaine Injection 1% with Epinephrine [1:100,000], Sandoz Pharma K.K., Tokyo, Japan) twice with saline. The 0.2% ropivacaine solution was used as supplied (Anapeine, Sandoz Pharma K.K., Tokyo, Japan). After exposure to the test solutions, the tissue fragments were immediately washed with saline and subjected to enzymatic digestion.

### Enzymatic digestion and cell isolation

The synovial fragments were digested with 0.4 mg/mL Liberase (Roche Diagnostics, Mannheim, Germany) at 37 °C for 3 h. Debris was then removed by passing the digest through a 70 µm cell strainer (Greiner Bio-One, Kremsmünster, Austria), and the filtrate was collected. The cells were washed with phosphate-buffered saline (PBS), and nucleated cell numbers and cell viability were evaluated using a Luna cell counter (Logos Biosystem, VA, USA).

### Primary culture and MSC expansion

The nucleated cells were plated in six 145 cm^2^ dishes at a density of 500 cells/cm^2^ in a growth medium consisting of α-modified essential medium (α-MEM; Thermo Fisher Scientific, Waltham, MA, USA), 10% fetal bovine serum (FBS; Thermo Fisher Scientific), and 1% antibiotic–antimycotic (Thermo Fisher Scientific). After 14 days of primary culture, cells from five dishes (with one dish reserved for morphological assessment) were detached using 0.25% trypsin and 1 mM EDTA (Thermo Fisher Scientific). The total MSC number per synovial weight was calculated and compared between the anesthetic groups and the saline group. This procedure was performed using samples from each of the eight donors.

Cell viability and nucleated cell number were evaluated immediately after enzymatic digestion, and the nucleated cell number was reassessed after 14 days of primary culture. For each donor and treatment condition, cells harvested from five culture dishes were pooled into a single tube, and cell viability and nucleated cell numbers were evaluated as a single combined measurement. Expanded synovial MSCs obtained after primary culture were used for both surface antigen analysis and subsequent multipotency assays.

### Surface antigens

Human synovial MSCs were detached using TrypLE™ Select Enzyme (1 × ; Thermo Fisher Scientific) and suspended in phosphate-buffered saline (PBS) containing 2% fetal bovine serum (FBS) and 5 mM ethylenediaminetetraacetic acid (EDTA). The cells were incubated for 30 min at 4 °C with the following antibodies: CD44–PE-Cy7, CD73–FITC, CD90–PE, CD105–APC, CD34–PerCP-Cy5.5, and CD45–APC-H7 (all diluted 1:200; BD Biosciences, San Jose, CA, USA). Corresponding isotype-matched antibodies were used as negative controls. The percentages of antigen-positive cells were analyzed using a BD FACSMelody™ cell sorter (BD Biosciences).

### Chondrogenesis

MSCs (2.5 × 10^5 cells) were transferred to a 15 mL polypropylene tube (Thermo Fisher Scientific) and centrifuged at 580 × g for 10 min at 4 °C. The resulting cell pellets were cultured under chondrogenic conditions in high-glucose Dulbecco’s Modified Eagle Medium (Thermo Fisher Scientific) supplemented with 1% insulin–transferrin–selenium (Corning, NY, USA), 50 µg/mL ascorbate-2-phosphate, 40 µg/mL L-proline (Sigma-Aldrich), 100 nM dexamethasone, 1% antibiotic–antimycotic, 10 ng/mL human transforming growth factor-β3 (Miltenyi Biotec Japan, Tokyo, Japan), and 500 ng/mL human bone morphogenetic protein 2 (Medtronic, Minneapolis, MN). After 3 weeks of pellet culture, cartilage pellet weights were measured. The pellets were then fixed in 10% neutral-buffered formalin, embedded in paraffin, and sectioned at 5 µm. Sections were stained with Safranin O (1B463; Chroma Gesellschaft Schmid & Co., Münster, Germany) and Fast Green (061–00,031; Wako) to visualize glycosaminoglycan distribution. The Safranin O–positive area rate (%) was quantified using ImageJ (National Institutes of Health, Bethesda, MD, USA), with identical threshold settings applied across all donors (Supplementary Fig. S1). For each of the eight donors, six replicates were analyzed, and the donor-specific median was used for statistical analyses^15^.

### Adipogenesis

Samples of the anesthetic-treated and saline control human synovial MSCs (100 cells) were plated in 10 cm dishes (Thermo Fisher Scientific) and cultured for 14 days to allow colony formation. After colony formation, the adherent cells were cultured for an additional 21 days in an adipogenic induction medium consisting of growth medium supplemented with 100 nM dexamethasone (Wako), 0.5 mM isobutylmethylxanthine (Sigma-Aldrich), 50 mM indomethacin (Sigma-Aldrich), 4.5 mg/mL D-( +)-glucose (Wako), and 10 µg/mL recombinant human insulin (Wako). The cells were then fixed with 10% neutral buffered formalin and stained with Oil Red O (Sigma-Aldrich), and colonies containing lipid droplets were manually counted as Oil Red O–positive colonies. Each dish was subsequently stained with 0.5% crystal violet to determine the total number of colonies, and the percentage of Oil Red O–positive colonies was calculated. Three replicates were analyzed per donor, and the donor-specific median was used for statistical analysis; this experiment was performed using samples from six donors^15^.

### Calcification

Samples of the anesthetic-treated and saline control human synovial MSCs (100 cells) were plated in 10 cm dishes and cultured for 14 days to allow colony formation. After colony formation, the adherent cells were cultured in a calcification-inducing medium consisting of growth medium supplemented with 50 µg/mL ascorbic acid 2-phosphate (Wako), 1 nM dexamethasone, and 10 mM β-glycerophosphate (Sigma-Aldrich). After 21 days of calcification induction, the cells were fixed with 10% neutral buffered formalin and stained with Alizarin Red (Sigma-Aldrich). The area percentage of Alizarin Red–stained mineralized matrix was quantified using ImageJ (National Institutes of Health). For each of the six donors, three replicates were evaluated, and the donor-specific median was used for statistical analysis^15^.

### Statistical analysis

Cell viability (%), nucleated cell number per synovial weight, cell number after 14 days of primary culture per synovial weight, and cartilage pellet weight were evaluated using six replicates per donor from eight donors. The rate of the Oil Red O–positive colony area to the total colony area and the rate of the Alizarin Red–positive area to the total colony area were evaluated using three replicates per donor from six donors.

Because all comparisons were performed using paired samples derived from the same donors, statistical comparisons were conducted between the saline and lidocaine groups and between the saline and ropivacaine groups using the Wilcoxon signed-rank test. A two-tailed p value < 0.05 was considered statistically significant. All statistical analyses were performed using GraphPad Prism version 6.07 (GraphPad Software, San Diego, CA, USA).

The sample size (n = 8 donors) chosen for this study was determined based on a previous study^12^, which demonstrated a marked reduction in cell viability in bone marrow–derived MSCs treated with 0.5% lidocaine (91 ± 1% in saline vs. 5 ± 2% in lidocaine), corresponding to a very large effect size (Cohen’s d > 3). Assuming a large effect size (Cohen’s d ≥ 1.2) for cell viability, which was one of the primary outcome measures in the present study, a two-tailed Wilcoxon signed-rank test with eight paired samples provided a statistical power greater than 80%. Power analysis was performed using G*Power version 3.1.9.6 (Heinrich Heine University Düsseldorf, Germany).

## Results

### Viability and cell yield immediately after enzymatic digestion

**Fig. 2:**
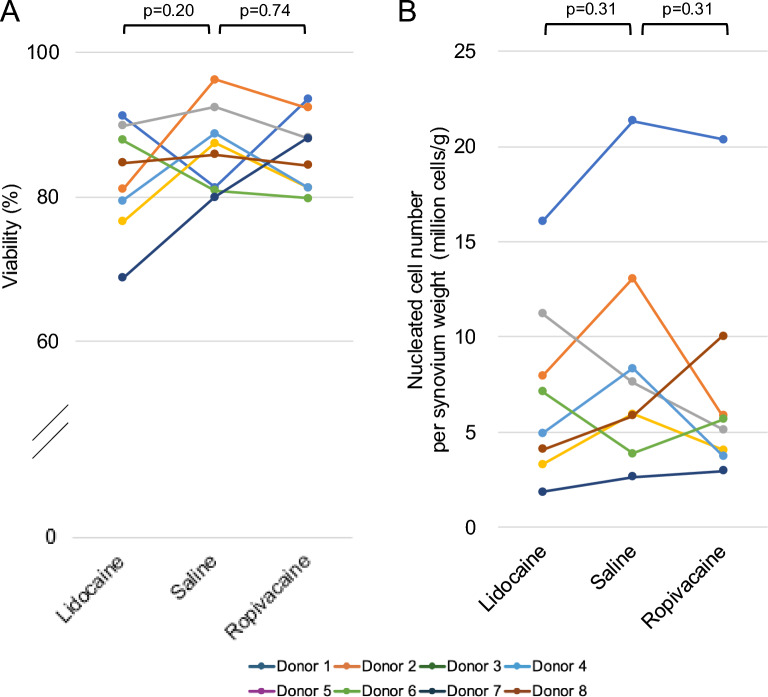
Effects of local anesthetics on synovial cell viability and yield after enzymatic digestion. Synovial tissue was divided into equal segments and immersed in saline, lidocaine, or ropivacaine. After enzymatic digestion, cell viability and nucleated cell numbers were assessed. The cells were then cultured for 14 days, and the synovial MSC yield per tissue weight was determined. (**A**) Cell viability. (**B**) Nucleated cell number per synovial weight. Data from the same donor are connected by lines of the same color. P values were calculated using the Wilcoxon signed-rank test by comparing each local anesthetic group with the saline control group.

Cell viability showed relatively small inter-donor variability across all treatment groups (Fig. 2A). No significant difference was observed between the saline and lidocaine groups (p = 0.20). Similarly, no significant difference was detected between the saline and ropivacaine groups (p = 0.74). In contrast, the nucleated cell numbers per synovial weight exhibited substantial inter-donor variability (Fig. 2B). However, no significant differences were observed between the saline and lidocaine groups (p = 0.31) or between the saline and ropivacaine groups (p = 0.31).

### Cell morphology and yield after a 14-day culture expansion

**Fig. 3:**
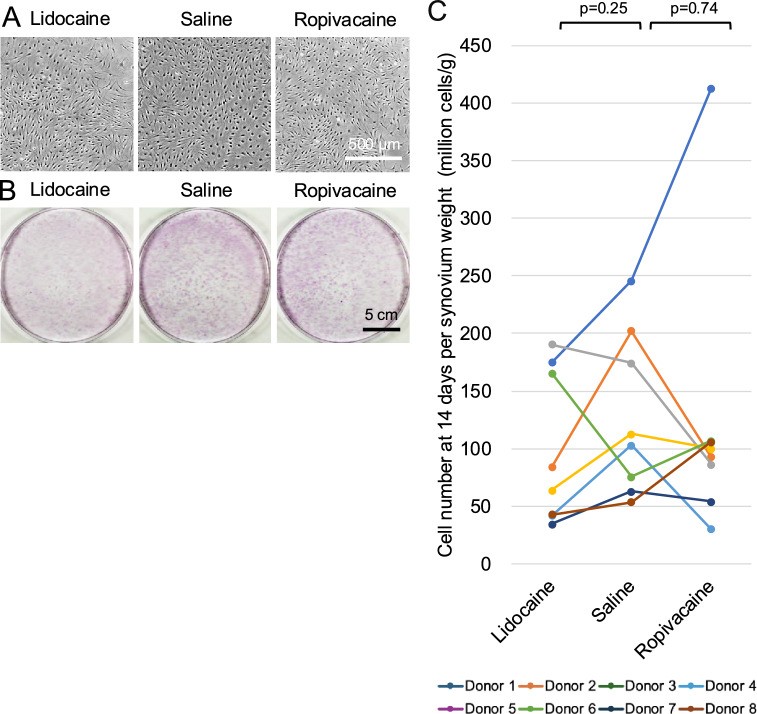
Effects of local anesthetics on synovial cell morphology and yield after replating. Synovial tissue fragments were exposed to lidocaine, saline, or ropivacaine, followed by enzymatic digestion. The released cells were replated and cultured for 14 days. (**A**) Morphology of synovial MSCs after primary culture. (**B**) Cell colonies visualized by crystal violet staining. (**C**) Synovial MSC number at 14 days per synovial weight. Data from the same donor are connected by lines of the same color. P values were calculated using the Wilcoxon signed-rank test comparing each local anesthetic group with the saline group.

After 14 days of culture, the synovial MSCs exhibited a spindle-shaped morphology with prominent nuclei. Cell morphology was comparable among the lidocaine, ropivacaine, and saline groups, with no observable differences in cell shape or cell density (Fig. 3A). Consistently, representative crystal violet–stained colonies showed no apparent differences in colony size or number among the three groups (Fig. 3B).

The cell yield after expansion showed substantial inter-donor variability (Fig. 3C). However, no significant differences in synovial MSC numbers per synovial weight were observed between the saline and lidocaine groups (p = 0.25) or between the saline and ropivacaine groups (p = 0.74).

**Fig. 4:**
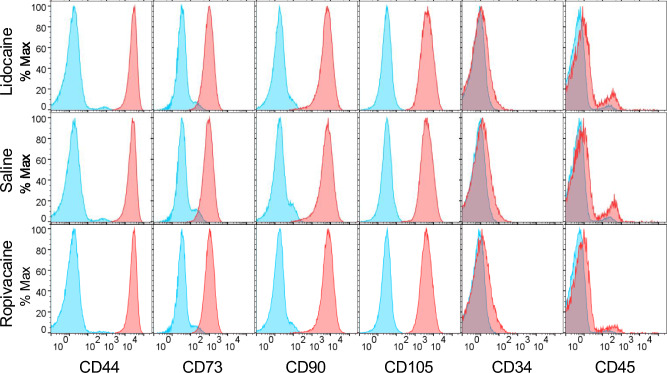
Flow cytometry profiles of synovial MSCs after exposure to local anesthetics. Synovial tissue fragments were exposed to lidocaine, saline, or ropivacaine, enzymatically digested, and expanded in monolayer culture. The expanded synovial MSCs were evaluated for surface marker expression. Blue histograms indicate unstained controls, and red histograms indicate antibody-stained cells. The x-axis represents fluorescence intensity, and the y-axis represents the percentage of cells.

Flow cytometry analysis demonstrated that synovial MSCs from all treatment groups consistently expressed MSC-associated surface markers (CD44, CD73, CD90, and CD105) and lacked expression of hematopoietic markers (CD34 and CD45). No notable differences in surface marker expression were detected among the treatment groups (Fig. 4).

### Chondrogenesis

**Fig. 5:**
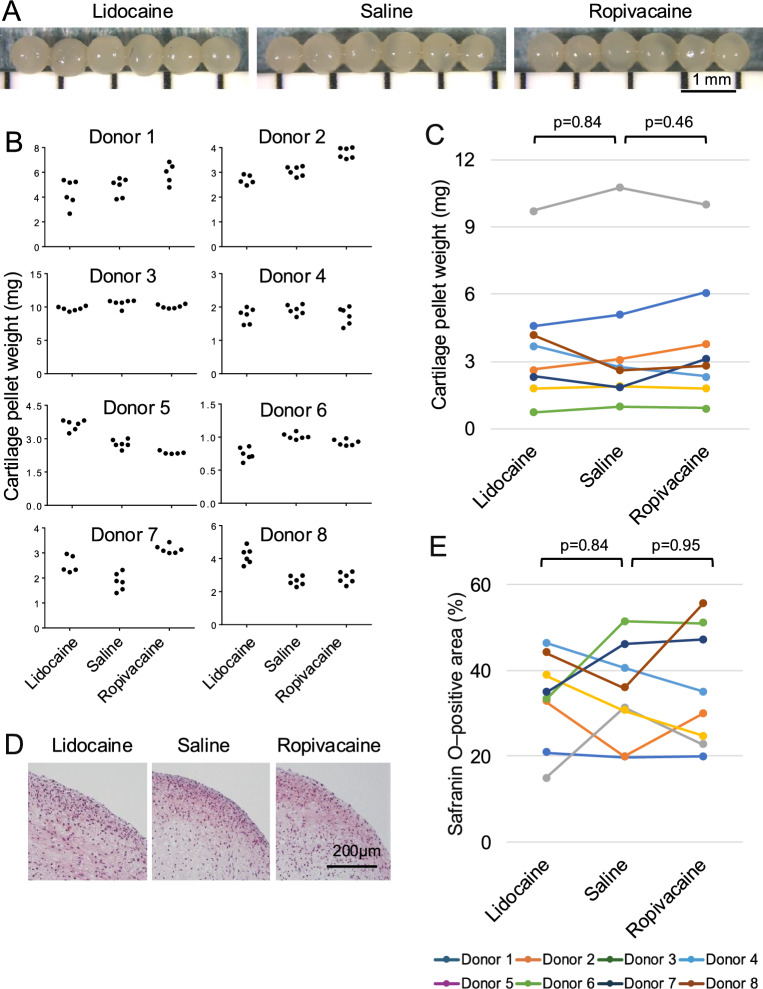
Effects of local anesthetics on chondrogenesis of synovial MSCs. Synovial tissue fragments were exposed to lidocaine, saline, or ropivacaine, enzymatically digested, and expanded in monolayer culture. The expanded synovial MSCs were then induced to undergo chondrogenic differentiation using a pellet culture system. (**A**) Macroscopic appearance of the cartilage pellets. All pellets derived from a representative donor (donor 1) are shown (n = 6). (**B**) Donor-level scatter plots of cartilage pellet weight under each treatment condition (six pellets per condition). (**C**) Median cartilage pellet weight across the eight donors. Data from the same donor are connected by lines of the same color. P values were calculated using the Wilcoxon signed-rank test comparing each local anesthetic group with the saline group. (**D**) Representative histological sections of cartilage pellets stained with Safranin O. (**E**) Median Safranin O–positive area rate (six pellets per donor) across the eight donors. Data from the same donor are connected by lines of the same color. P values were calculated using the Wilcoxon signed-rank test comparing each local anesthetic group with the saline group.

All treatment groups successfully formed spherical cartilage pellets (Fig. 5A). For each donor, the cartilage pellet weights were relatively consistent across the six replicates per treatment group, indicating good reproducibility of the chondrogenic differentiation protocol (Fig. 5B). In contrast, substantial inter-donor variability was observed regarding the pellet weights among the eight donors examined (Fig. 5C). Despite this variability, no significant differences were detected between the saline and lidocaine groups (p = 0.84) or between the saline and ropivacaine groups (p = 0.46). Histological analysis demonstrated that all cartilage pellets were positive for Safranin O staining, indicating glycosaminoglycan deposition (Fig. 5D). Consistent with the macroscopic findings, no significant differences were detected in the Safranin O–positive area rates between the saline and lidocaine groups (p = 0.84) or between the saline and ropivacaine groups (p = 0.95) (Fig. 5E and Supplementary Fig. S2).

### Adipogenesis

**Fig. 6:**
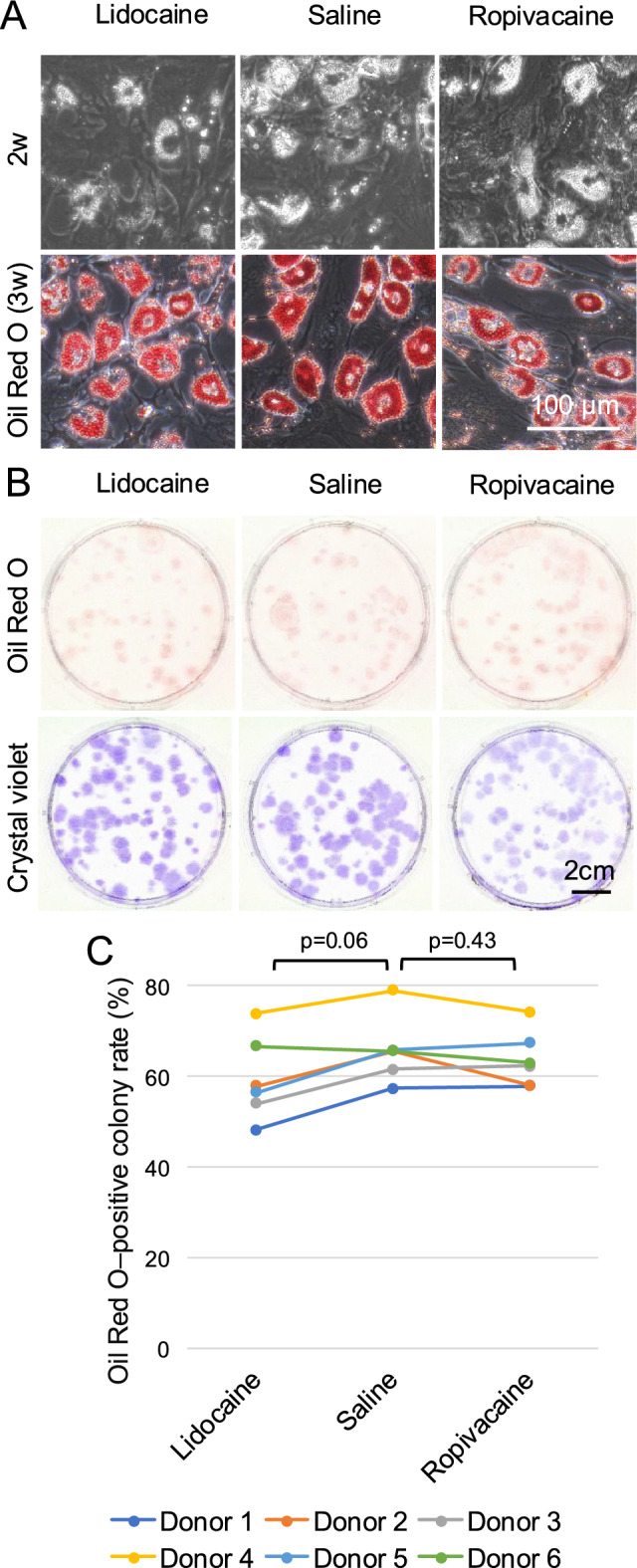
Effects of local anesthetics on adipogenesis of synovial MSCs. Synovial tissue fragments were exposed to lidocaine, saline, or ropivacaine, enzymatically digested, and expanded in monolayer culture. The expanded synovial MSCs were cultured to form colonies and subsequently induced to undergo adipogenic differentiation. Cultures were stained with Oil Red O to identify adipogenic colonies, followed by crystal violet staining to determine the proportion of Oil Red O–positive colonies. (**A**) Representative phase-contrast images before induction (2 weeks) and Oil Red O–stained adipogenic cultures after 3 weeks of induction. (**B**) Macroscopic images of Oil Red O–stained adipogenic colonies (upper panels) and corresponding crystal violet–stained total colonies (lower panels). (**C**) Median Oil Red O–positive colony rate (%) across six donors. Data from the same donor are connected by lines of the same color. P values were calculated using the Wilcoxon signed-rank test comparing each local anesthetic group with the saline group.

Microscopic examination of the colonies revealed minimal lipid droplet formation at 2 weeks, whereas prominent intracellular lipid droplets were clearly visualized by Oil Red O staining at 3 weeks across all treatment groups (Fig. 6A). Oil Red O–positive colonies were observed under all conditions (Fig. 6B). The Oil Red O–positive colony rate showed relatively low inter-donor variability compared with the other evaluated parameters, ranging from approximately 45% to 80% across the six donors (Fig. 6C and Supplementary Fig. S3). No significant differences were observed between the saline and lidocaine groups (p = 0.06) or between the saline and ropivacaine groups (p = 0.43).

### Calcification

**Fig. 7:**
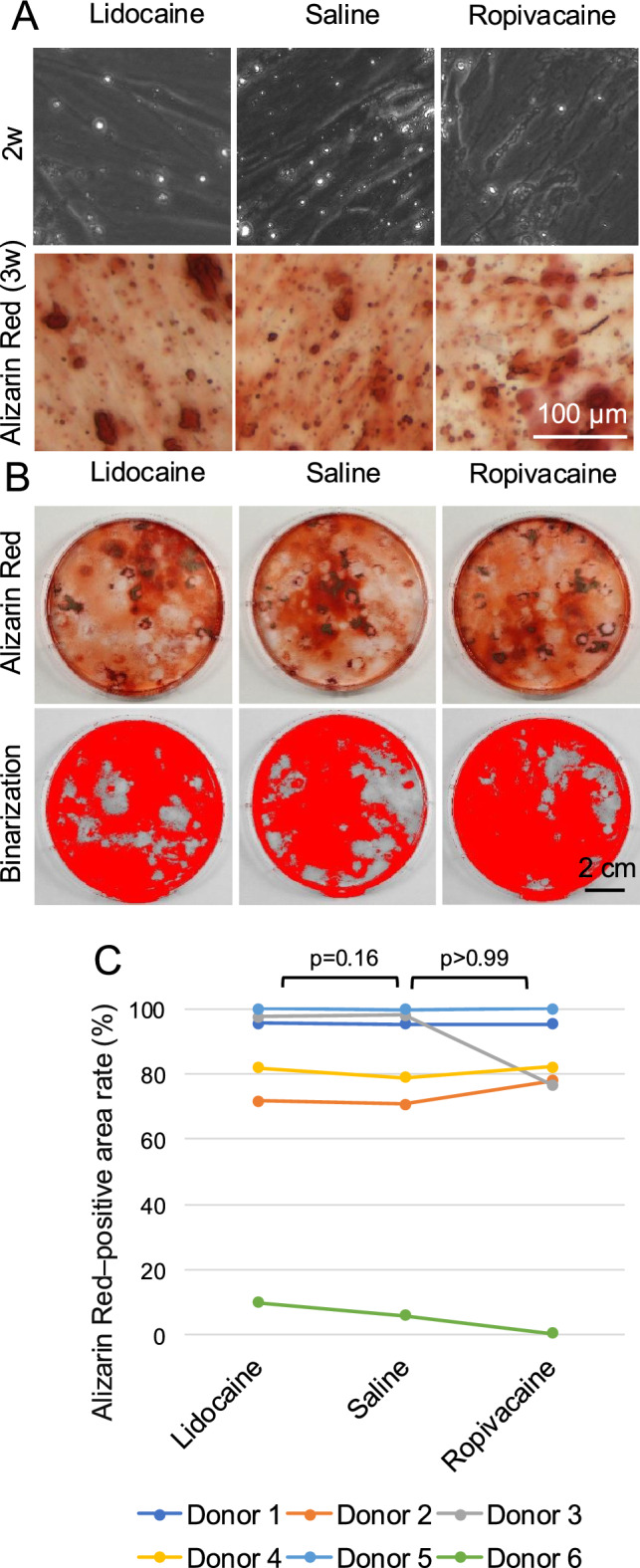
Effects of local anesthetics on calcification of synovial MSCs. Synovial tissue fragments were exposed to lidocaine, saline, or ropivacaine, enzymatically digested, and expanded in monolayer culture. The expanded synovial MSCs were cultured to form colonies and subsequently induced to undergo calcification. (**A**) Representative phase-contrast images at 2 weeks after the start of calcification induction and Alizarin Red–stained cultures at 3 weeks. (**B**) Representative culture dishes stained with Alizarin Red (upper panels) and corresponding binarized images used for quantification of Alizarin Red–positive areas (lower panels). (**C**) Median Alizarin Red–positive area rate (%) across six donors (three replicates per donor). Data from the same donor are connected by lines of the same color. P values were calculated using the Wilcoxon signed-rank test comparing each local anesthetic group with the saline group.

At 2 weeks, the colonies displayed round refractile granules consistent with early-stage mineral deposition under phase-contrast microscopy, and subsequent Alizarin Red staining at 3 weeks confirmed calcified nodule formation in the same cultures (Fig. 7A). Representative whole-dish images after Alizarin Red staining were used to determine the distribution of calcified areas (Fig. 7B). The corresponding binarized images were used for quantitative analysis, which revealed substantial inter-donor variability, as shown in both the donor-connected plot and the individual donor data (Fig. 7C and Supplementary Fig. S4). Despite this variability, no significant differences were observed in the Alizarin Red–positive area rates between the saline and lidocaine groups (p = 0.16) or between the saline and ropivacaine groups (p > 0.99).

## Discussion

Local anesthetic use, synovial tissue harvesting, and successful synovial MSC culture are key elements in the clinical translation of regenerative therapies for joint diseases. In this study, we addressed a clinically relevant question arising from the increasing use of minimally invasive synovial tissue collection procedures. Specifically, we examined whether exposure of synovial tissue to local anesthetics adversely affects the yield and functional properties of synovial MSCs derived later from the exposed tissues. Our results demonstrate that brief exposure of synovial tissue to clinically relevant concentrations of lidocaine or ropivacaine does not compromise subsequent MSC viability, expansion capacity, or multilineage differentiation potential. These findings provide experimental evidence supporting the safe use of local anesthetics during synovial tissue collection and reinforce the feasibility of synovial MSC–based regenerative therapies.

To our knowledge, this study is the first to specifically investigate the effects of local anesthetics on synovial MSCs. Previous studies have predominantly focused on bone marrow–derived MSCs^11,12,16^ and adipose-derived MSCs^17,18^, largely using monolayer culture systems. These previous investigations have demonstrated that susceptibility to local anesthetic–induced cytotoxicity differs depending on the MSC source. In particular, adipose-derived MSCs appear more vulnerable to cell death when exposed as cell culture monolayers to relatively low anesthetic concentrations, potentially reflecting increased structural fragility during adipogenic differentiation^19^. In contrast to these previous approaches, we exposed synovial MSCs while they were still within their native tissue environment rather than as isolated cells in monolayer culture.

The use of this tissue-based exposure model may partly explain why no cytotoxic effects were observed in the MSCs from synovial tissues exposed to clinically relevant anesthetic conditions. Histologically, the synovium consists of an inner lining layer composed of one to three cell layers and a sub-synovial region primarily composed of loose connective tissue. The lining layer contains fibroblast-like synoviocytes that produce hyaluronic acid, whereas the sub-synovial region comprises a fibrous extracellular matrix rich in collagens type III, IV, V, and VI, as well as laminin and fibronectin. Importantly, the synovial MSCs are predominantly located within this sub-synovial region^20,21^.

These structural features suggest that the extracellular matrix of the synovial tissue may be able to mitigate the cytotoxic effects of local anesthetic exposure. Supporting this concept, previous studies on chondrocytes have shown that co-administration of hyaluronic acid suppresses lidocaine-induced apoptosis, suggesting an attenuation of local anesthetic–induced cytotoxicity by high-molecular-weight matrix components^22^. Similarly, Breu et al. demonstrated that bone marrow–derived MSCs, which exhibit marked cell death in undifferentiated monolayer cultures, show reduced cytotoxicity when maintained as cartilage-differentiated aggregates in three-dimensional culture systems^23^. The protective effects observed in these 3D aggregates were attributed to physical buffering by the extracellular matrix and activation of anti-apoptotic signaling pathways via integrin-mediated mechanisms^24^.

In the present study, synovial tissue was minced prior to anesthetic exposure, creating a partial disruption of the native spatial organization of the synovial layers. Nevertheless, this experimental approach did not necessarily underestimate clinical anesthetic exposure. In clinical practice, particularly during ultrasound-guided synovial tissue harvesting, local anesthetics are frequently administered both intra-articularly and directly into the synovial tissue to ensure sufficient analgesia. Therefore, synovial MSCs may be exposed to local anesthetics in close proximity within the tissue itself. In this context, direct exposure of synovial tissue fragments to local anesthetics in the present study may represent a conservative approximation of the actual clinical situation rather than a milder exposure condition, and may explain the preserved viability and functional properties of the synovial MSCs observed in this study.

The cytotoxic effects of local anesthetics on MSCs in monolayer culture systems have been well documented. Lucchinetti et al. reported that lidocaine and ropivacaine induced cell cycle arrest in mouse bone marrow–derived MSCs at the G0/G1 phase through inhibition of the NF-κB–ICAM-1 signaling pathway^16^. Similarly, Dregalla et al. showed that exposure of human bone marrow–derived MSCs to various local anesthetics reduced WST-1 absorbance, reflecting impaired mitochondrial dehydrogenase activity and compromised cellular metabolic function, ultimately leading to reduced cell viability and proliferative capacity^12^. However, other studies have reported differences in the cytotoxic potential between lidocaine and ropivacaine^11,12^.

Both lidocaine and ropivacaine are amide-type local anesthetics that block sodium channels to inhibit nerve signal transmission^25,26^, but they differ in their physicochemical properties and intracellular distributions. Lidocaine is relatively water soluble and readily enters the cytoplasm, where it can impair mitochondrial function and induce apoptosis. In contrast, ropivacaine is more lipophilic and tends to localize within cell membranes, resulting in less intracellular damage^11^. Multiple studies have reported milder cellular toxicity with ropivacaine than with lidocaine in assays evaluating cell survival, energy metabolism, and protein expression^11–14^,^16,27^. Although neither lidocaine nor ropivacaine showed overt cytotoxicity in the present study, this investigation was not designed for a direct head-to-head comparison between these agents. The mechanistic differences should be addressed in future investigations.

Our flow cytometry analysis confirmed that synovial MSCs from all treatment groups consistently expressed canonical MSC surface markers (CD44, CD73, CD90, and CD105) while exhibiting minimal expression of hematopoietic markers (CD34 and CD45). These phenotypic profiles are consistent with the criteria established for bone marrow–derived MSCs^28^ and indicate that exposure to local anesthetics did not alter the MSC identity. Similarly, anesthetic exposure did not prevent the MSCs from developing chondrogenic, adipogenic, or calcification potentials.

Chondrogenesis was primarily evaluated using cartilage pellet weight as an outcome measure. Previous in vitro studies of MSC chondrogenesis have demonstrated that pellet size and weight increase in parallel with extracellular matrix synthesis, while the DNA yield per pellet decreases. Radioactivity per DNA in cells prelabeled with ^3^H-thymidine remains stable during chondrogenic differentiation, indicating that pellet enlargement predominantly reflects increased matrix production rather than enhanced cell proliferation^29^. Accordingly, cartilage pellet weight largely reflects the amount of cartilage matrix produced per cell and has been widely used as a surrogate marker of in vitro chondrogenesis in MSC populations^4,15,30^.

Several previous studies have examined the effects of local anesthetics on chondrogenic differentiation at the gene and protein levels. For example, Kim et al. reported that a 60 min treatment of human dental pulp–derived MSCs with 1% lidocaine significantly reduced COL2A1 and SOX9 mRNA expression^31^. Similarly, Breu et al. observed decreased glycosaminoglycan and type II collagen content in bone marrow–derived MSCs following exposure of monolayer cultures to anesthetics^23^. The discrepancies between these findings and the present results may reflect differences in experimental design, particularly the application of the anesthetics to isolated cells in monolayer culture versus MSCs within intact tissues.

In the present study, adipogenesis and calcification were assessed using Oil Red O–positive colony rates and Alizarin Red–positive area rates, respectively. The lack of any significant differences between saline-treated controls and either anesthetic group indicated that neither lidocaine nor ropivacaine impaired the multilineage differentiation potential of the synovial MSCs. Preservation of this multipotency is a fundamental requirement for clinical application in regenerative medicine. Therefore, an important question for future investigation is whether the tissue-specific resistance to local anesthetic–induced cytotoxicity observed in this native tissue–based model also applies to synovial MSCs cultured as isolated monolayers. Synovial MSCs may exhibit different susceptibility to local anesthetics when removed from their native extracellular matrix and cultured under conventional monolayer conditions. Comparative studies examining synovial MSCs alongside other MSC populations under identical monolayer culture conditions may help clarify intrinsic cellular mechanisms underlying differential susceptibility to anesthetic exposure.

This study has several limitations. First, the synovial tissue exposure protocol does not precisely replicate clinical practice, where lidocaine is directly injected into the synovium under ultrasound guidance before tissue retrieval. Second, the lidocaine formulation used in this study also contained epinephrine, which may influence cytotoxicity and should be considered when comparing results with previous studies. Third, despite our efforts to distribute synovial tissue fragments evenly, tissue heterogeneity—such as the proportion of adipose tissue—within individual treatment groups was unavoidable. Fourth, chondrogenesis was primarily assessed using cartilage pellet weight and Safranin O histology. Although these are widely accepted surrogate measures, more detailed analyses—such as type II collagen quantification or normalization of matrix production to DNA content—would provide a more comprehensive assessment.

In conclusion, this study demonstrates that brief exposure of synovial tissue to clinically relevant concentrations of lidocaine or ropivacaine does not adversely affect the viability, expansion capacity, or multilineage differentiation potential of synovial MSCs. These findings support the safe use of local anesthetics during minimally invasive synovial tissue collection and provide practical guidance for procedural design in synovial MSC–based regenerative therapies.

## Supplementary Information

Supplementary Information.

## Data Availability

The datasets used and/or analyzed during the current study are available from the corresponding author on reasonable request.

## Abbreviations

α-MEM: α-modified essential medium
EDTA: Ethylenediaminetetraacetic acid
FACS: Fluorescence-activated cell sorting
GAG: Glycosaminoglycan
LA: Local anesthetic
MSCs: Mesenchymal stem cells
OA: Osteoarthritis
PBS: Phosphate-buffered saline
