# Supplementary material for: Effects of local anesthetics on yield and differentiation of synovial mesenchymal stem cells
Source: Sci Rep. 2026 Jan 16;16:5557. doi: 10.1038/s41598-026-36025-z (PMC12891527; doi:10.1038/s41598-026-36025-z)
Supplement: Supplementary file 1 — Supplementary Information. [file 41598_2026_36025_MOESM1_ESM.pptx]

## Slide 1
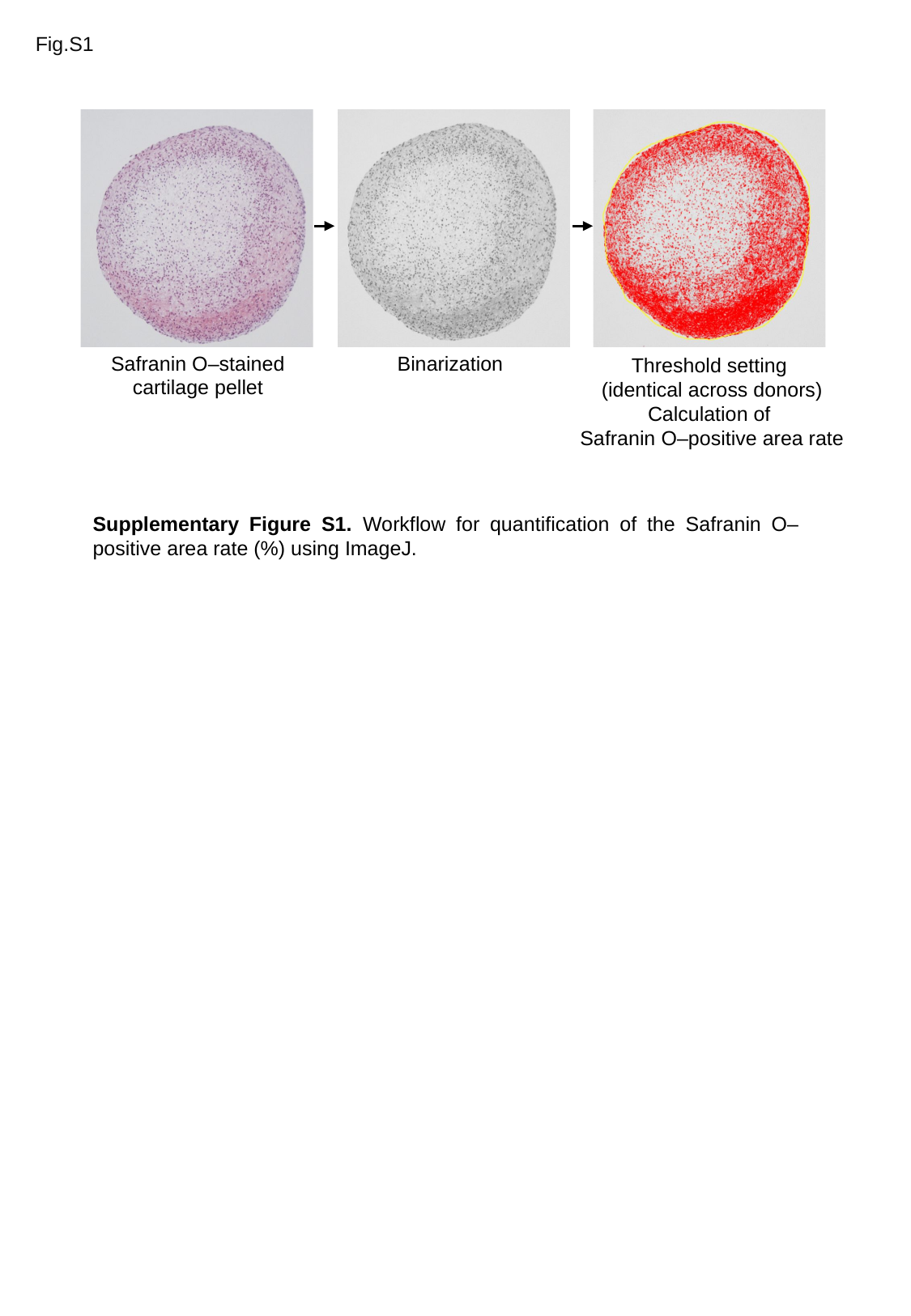

Fig.S1
Safranin O–stained cartilage pellet
Binarization
Threshold setting
(identical across donors)
Calculation of
Safranin O–positive area rate
Supplementary Figure S1. Workflow for quantification of the Safranin O–positive area rate (%) using ImageJ.

## Slide 2
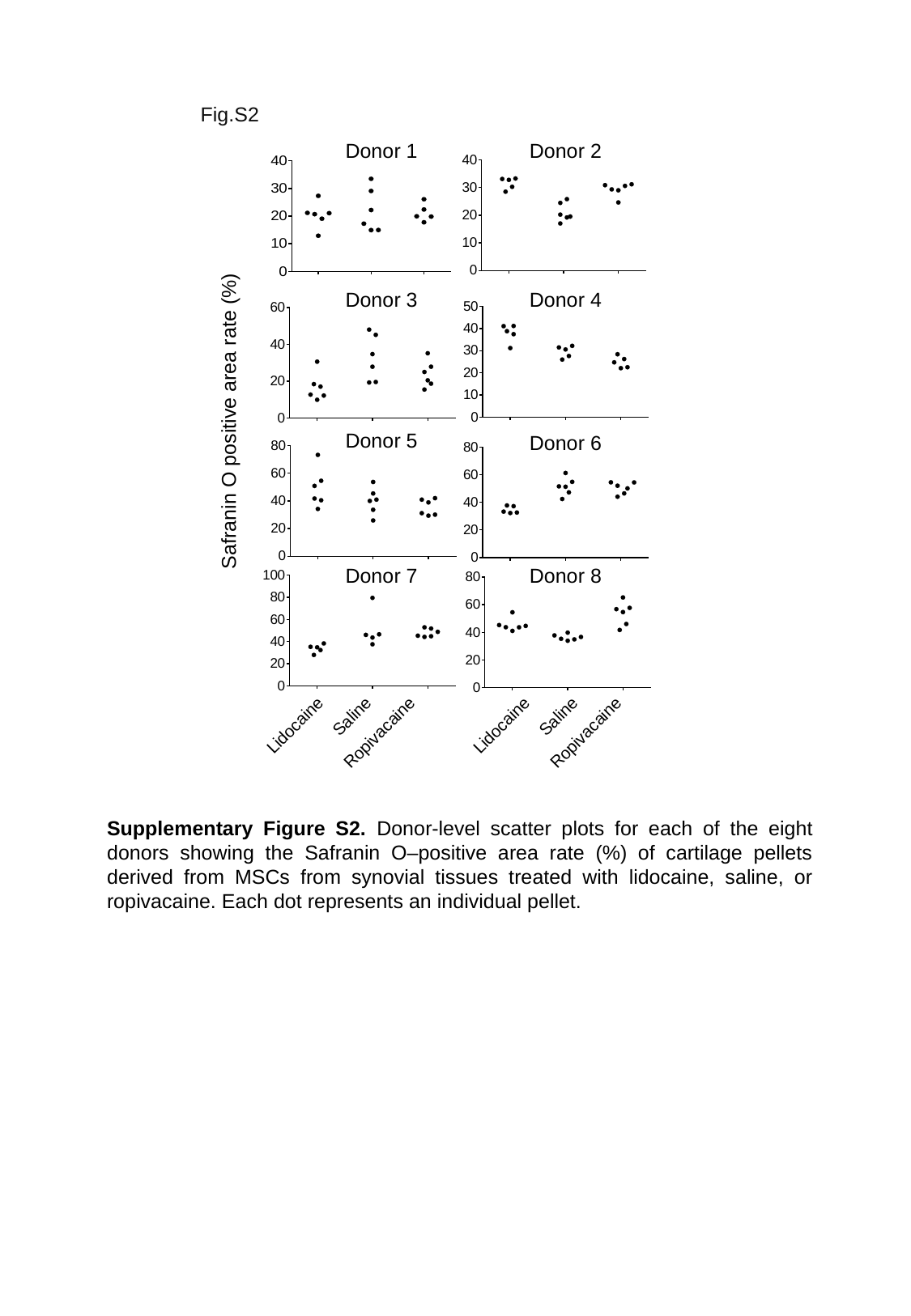

Fig.S2
Donor 1
Donor 2
Safranin O positive area rate (%)
Donor 3
Donor 4
Donor 5
Donor 6
Donor 7
Donor 8
Saline
Saline
Lidocaine
Lidocaine
Ropivacaine
Ropivacaine
Supplementary Figure S2. Donor-level scatter plots for each of the eight donors showing the Safranin O–positive area rate (%) of cartilage pellets derived from MSCs from synovial tissues treated with lidocaine, saline, or ropivacaine. Each dot represents an individual pellet.

## Slide 3
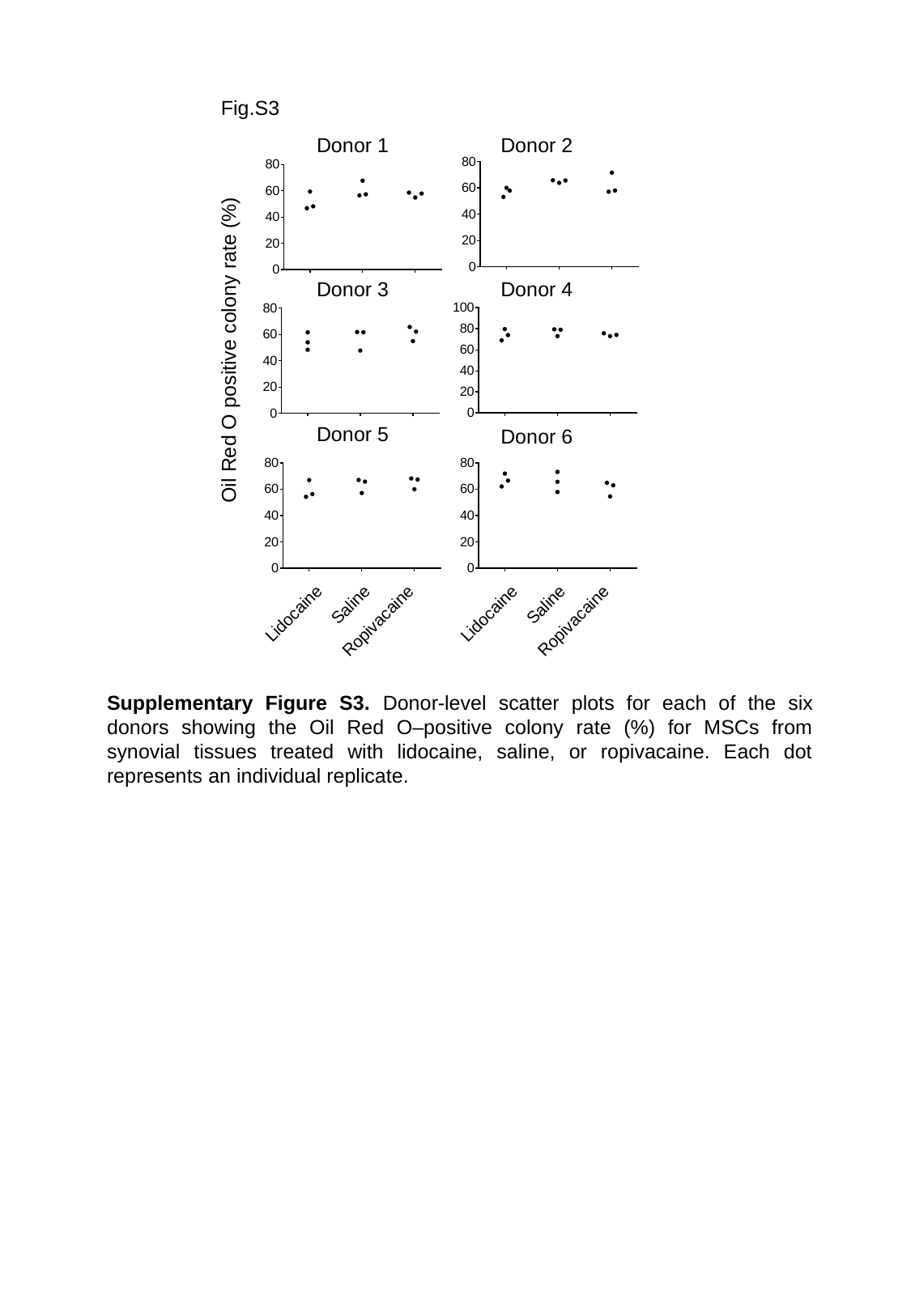

Fig.S3
Donor 1
Donor 2
Oil Red O positive colony rate (%)
Donor 3
Donor 4
Donor 5
Donor 6
Saline
Saline
Lidocaine
Lidocaine
Ropivacaine
Ropivacaine
Supplementary Figure S3. Donor-level scatter plots for each of the six donors showing the Oil Red O–positive colony rate (%) for MSCs from synovial tissues treated with lidocaine, saline, or ropivacaine. Each dot represents an individual replicate.

## Slide 4
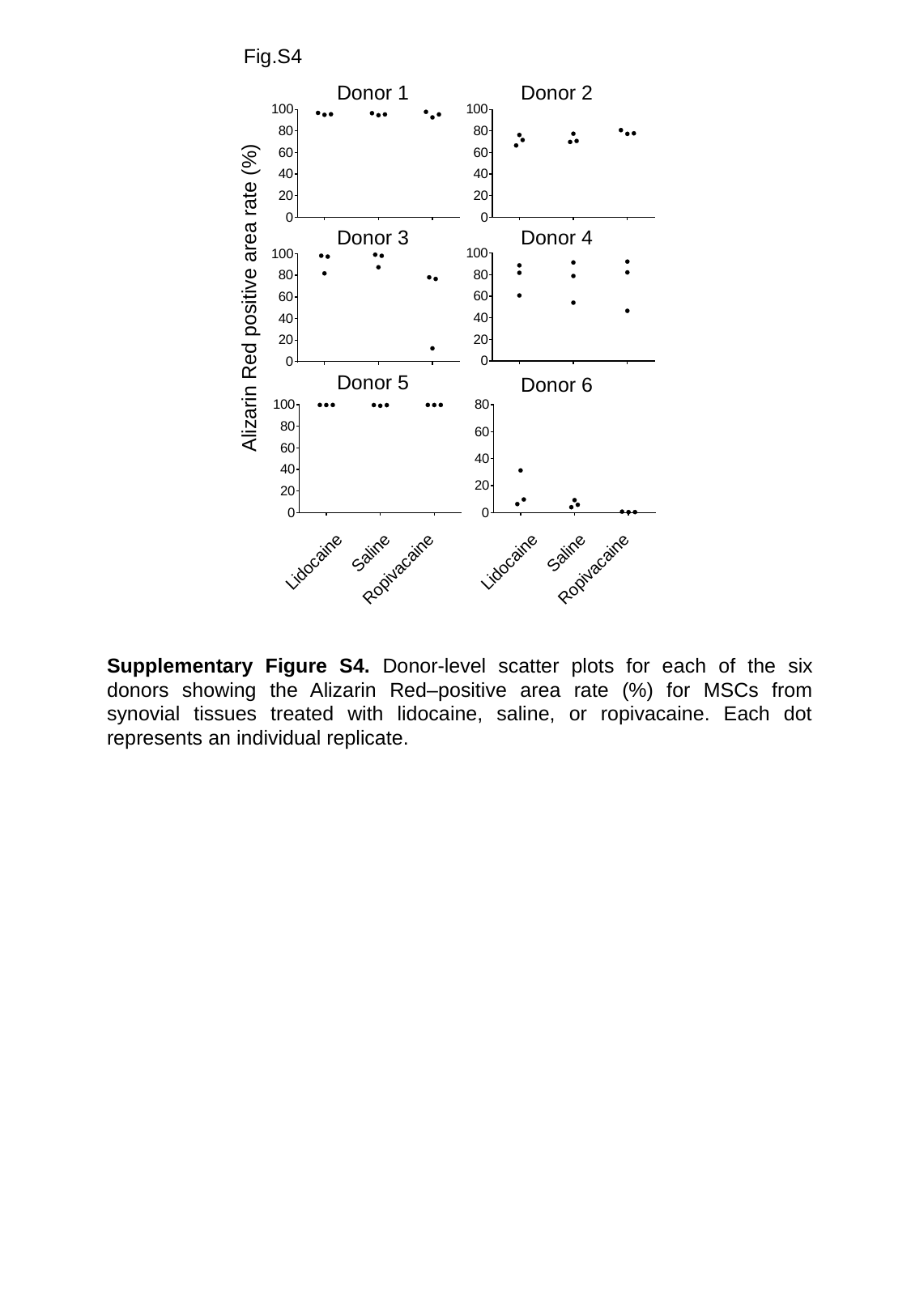

Fig.S4
Donor 1
Donor 2
Alizarin Red positive area rate (%)
Donor 3
Donor 4
Donor 5
Donor 6
Saline
Saline
Lidocaine
Lidocaine
Ropivacaine
Ropivacaine
Supplementary Figure S4. Donor-level scatter plots for each of the six donors showing the Alizarin Red–positive area rate (%) for MSCs from synovial tissues treated with lidocaine, saline, or ropivacaine. Each dot represents an individual replicate.
